# iTRAQ-based proteomic profiling reveals protein alterations after traumatic brain injury and supports thyroxine as a potential treatment

**DOI:** 10.1186/s13041-021-00739-0

**Published:** 2021-01-27

**Authors:** Zhongxiang Zhang, Jiangtao Yu, Pengcheng Wang, Lian Lin, Ruining Liu, Rong Zeng, Haoli Ma, Yan Zhao

**Affiliations:** 1 Emergency Center, Zhongnan Hospital of Wuhan University, Wuhan, 430071 China; 2 Hubei Clinical Research Center for Emergency and Resuscitation, Zhongnan Hospital of Wuhan University, Wuhan, 430071 China; 3 Department of Biological Repositories, Zhongnan Hospital of Wuhan University, Wuhan, 430071 China

**Keywords:** Traumatic brain injury, Quantitative proteomics, Mass spectrometry (MS), Parallel reaction monitoring (PRM), Thyroxine, Transthyretin, Rat cortex

## Abstract

Traumatic brain injury (TBI) is a primary cause of disability and death across the world. Previously, RNA analysis was widely used to study the pathophysiological mechanisms underlying TBI; however, the relatively low correlation between the transcriptome and proteome revealed that RNA transcription abundance does not reliably predict protein abundance, which led to the emergence of proteomic research. In this study, an iTRAQ proteomics approach was applied to detect protein alterations after TBI on a large scale. A total of 3937 proteins were identified, and 146 proteins were significantly changed after TBI. Moreover, 23 upregulated proteins were verified by parallel reaction monitoring (PRM), and fold changes in 16 proteins were consistent with iTRAQ outcomes. Transthyretin (Ttr) upregulation has been demonstrated at the transcriptional level, and this study further confirmed this at the protein level. After treatment with thyroxine (T4), which is transported by Ttr, the effects of T4 on neuronal histopathology and behavioral performance were determined in vivo (TBI + T4 group). Brain edema was alleviated, and the integrity of the blood brain barrier (BBB) improved. Escape latency in the Morris water maze (MWM) declined significantly compared with the group without T4 treatment. Modified neurological severity scores (mNSS) of the TBI + T4 group decreased from day 1 to day 7 post-TBI compared with the TBI + saline group. These results indicate that T4 treatment has potential to alleviate pathologic and behavioral abnormalities post-TBI. Protein alterations after T4 treatment were also detected by iTRAQ proteomics. Upregulation of proteins like Lgals3, Gfap and Apoe after TBI were reversed by T4 treatment. GO enrichment showed T4 mainly affected intermediate filament organization, cholesterol transportation and axonal regeneration. In summary, iTRAQ proteomics provides information about the impact of TBI on protein alterations and yields insight into underlying mechanisms and pathways involved in TBI and T4 treatment. Finally, Ttr and other proteins identified by iTRAQ may become potential novel treatment targets post-TBI.

## Introduction

Traumatic brain injury (TBI) is caused by an external force damaging the brain and is a major cause of disability and death in the United States [1]. In China, though the mortality rate due to TBI has declined in recent years, it remains relatively high, affecting 12.99 per 100,000 people in 2013 [2]. The economic burden of TBI is enormous, especially for people of low socioeconomic status, and these people happen to suffer from TBI at a higher prevalence [3, 4]. The pathophysiology of TBI-related injury is divided into primary and secondary brain injury. Primary brain injury is caused directly by the impact. Secondary brain injury is characterized by a series of pathophysiological processes including electrolyte imbalance [5], mitochondrial dysfunction [6], neuroinflammation [7], brain edema [8], and cerebral vascular injury [9]. Despite some progress, the complexity and interactions within these pathways have not been fully illuminated. Thus, current treatment of TBI is still primarily based on symptomatic treatment, focusing on acute management instead of specific medicinal therapy targeting the pathways involved in post-TBI pathophysiology [10]. Hence, further research is required to elucidate the mechanisms underlying TBI and identify suitable therapeutic targets in order to determine more precise and potent clinical treatment methods.

Over the past few years, researchers have increasingly focused on complicated pathways underlying TBI and discovered some effective brain protection strategies. Previous research has revealed that the nuclear factor-erythroid 2 related factor 2 (Nrf2) pathway protects TBI-affected rats from oxidative stress and TBI-induced apoptosis, and some pharmaceutical intervention targeting this pathway has been proven effective in animal experiments [11–14]. NF-kappa b-mediated signaling has been reported to have important associations with neuronal damage and neuroinflammation after TBI, and it may be a potential clinical target for some medications [15–18]. The PI3K/AKT pathway, associated with autophagy, also plays a vital role in neuronal protection from apoptosis after TBI [19–21]. Reducing JAK/STAT pathway activation after experimental TBI has been reported to improve vestibular motor recovery via regulation of the γ-aminobutyric acid (GABA) type A receptor [22]. In addition, the mitogen-activated protein kinase (MAPK) pathway [23, 24], glycogen synthase kinase-3 (GSK3) pathway [25–27], and AMP-activated kinase (AMPK) pathway [28] are all considered to be possible therapeutic approaches.

Despite continuing efforts, the mechanisms involved in TBI are still not entirely clear. Currently, proteomics is popular in life science research to detect protein alterations and identify novel targets. Great advances in quantitative proteomics have been made, and proteomics has become large-scale and enabled detailed functional characterization of certain biochemical processes [29, 30]. Recent research has revealed that the average correlation between the transcriptome and proteome is less than 30%, indicating that mRNA transcript abundance cannot reliably predict protein abundance. This inconsistency may be due to post-transcriptional modifications such as methylation and alternative splicing [31, 32], which makes proteomic research necessary.

A combination of liquid chromatography-tandem mass spectrometry (LC–MS/MS) and isobaric tags for relative and absolute quantitation (iTRAQ) has become an effective and widely used tool in quantitative proteomics studies, which is highly sensitive and repeatable [33]. Proteomic research on neural diseases has mainly focused on neurodegenerative diseases, such as Alzheimer’s disease and Huntington’s disease [34–36], epilepsy [37], narcolepsy [38], and ischemic cerebral disease [39, 40]. As for TBI, proteomics has been primarily applied to discover new biomarkers [41, 42]. In particular, Cheng et al. found that targeted temperature management (TTM) prevented TBI-induced neuronal necrosis, neuronal death, and brain edema, and some altered proteins (i.e., plasminogen, antithrombin III, and transthyretin) were identified [43]. Wu et al. detected protein changes in the traumatically injured hippocampus and found that pathways associated with global energy metabolism were significantly downregulated [44]. Song et al. determined that upregulation of the cAMP pathway was associated with mild TBI [45]. Overall, analyses of proteomic changes underlying TBI have been rarely reported. Particularly, results of the aforementioned research were not exactly consistent, because the samples were from different species and origins (Human blood, cells, hippocampus, brain tissue covering the injury or temporal globe in rats), and the labels were also different (iTRAQ or TMT). Besides, animals have individual differences. The different approaches like CCI, fluid induced percussion and blast were used for TBI models, which could also influence proteomic outcome. This study intends to detect the proteomic profile in cortex of rats and to identify key proteins involved in TBI pathophysiology.

In this study, LC–MS/MS combined with iTRAQ was utilized to identify differentially expressed proteins comparing rats in a TBI group with those in a sham group. Bioinformatic methods such as gene ontology (GO), the Kyoto Encyclopedia of Gene and Genomes (KEGG), and protein–protein interaction (PPI) analyses were used to analyze the biological processes, molecular functions, and interaction networks of these proteins. To confirm iTRAQ results, parallel reaction monitoring (PRM) was used to verify changes of proteins of interest. Using histopathology and behavioral measurements of the rats, the effects of thyroxine (T4), which is transported by transthyretin (Ttr), on pathophysiological features and neurological function after TBI were validated. This work may lead to a more comprehensive understanding of the mechanisms underlying TBI and the discovery of new pathways and molecules that can be used clinically.

## Methods

### Animals

A total of 76 male Sprague-Dawley rats (aged 10 weeks, weighing 250–300 g) were purchased from Vital River Laboratory Animal Technology Co. Ltd. (Vital River, Beijing, China), kept at room temperature (RT) for a 12 h light/dark cycle, and had free access to food and water. The rats were kept in the Animal Experimental Center of Zhongnan Hospital of Wuhan University, China, for 1 week prior to surgery. All procedures were approved by the Animal Experiment Center and Ethics Committee of Zhongnan Hospital of Wuhan University and were performed under the National Institutes of Health Guide for the Care and Use of Laboratory Animals of China.

### Cortical contusion impact model and experimental groups

The rats were randomly divided into two groups (sham group and TBI group), each of which contained 38 rats. A weight-drop device was applied to produce the cortical contusion impact (CCI), which was used to impose moderate TBI in previous studies [46, 47]. Rats were anesthetized with 1% pentobarbital (30 mg/kg) by intraperitoneal (i.p.) injection. A midline scalp incision was then performed under sterile conditions. After exposing the skull, a hole (5 mm diameter) was drilled on the right, equidistant between the lambda and bregma (2 mm), exposing the dura mater. A 50 g weight fell vertically from a height of 25 cm, striking the exposed brain tissue and causing moderate TBI. Rats in the sham group underwent identical surgical craniotomy without the contusion. Eight rats were divided into two groups (sham and TBI, n = 4).  24 h after surgery, the 8 rats were sacrificed and 3 mm of undamaged brain cortex around the damaged tissue was surgically collected for iTRAQ analysis. Another 8 rats were also divided into two groups (sham and TBI, n = 4) to verify the results of iTRAQ by PRM analysis. Moreover, another 12 rats (sham + saline, TBI + saline, and TBI + T4, n = 4) were sacrificed and cortices were collected after behavioral experiments for iTRAQ analysis. The tissue for sequencing was stored in a refrigerator at − 80 °C.

### Protein extraction

Cortical samples were transferred into low protein binding tubes (1.5 mL, Eppendorf, Hamburg, Germany) and lysed with 300 µL of lysis buffer (Beyotime, Shanghai, China) and 1 mM phenylmethyl sulfonyl fluoride (PMSF, Amresco, Solon, Ohio, USA). Next, the samples were homogenized and sonicated on ice to be lysed. The parameters were set to 1 s intervals (3 min total) and a power of 80 W. After being sonificated, the samples were centrifuged at 12,000×*g* for 10 min at RT, and supernatants were collected. Samples were centrifuged twice to exclude precipitation completely. Protein concentration was measured by bicinchoninic acid (BCA) assay (Thermo Fisher Scientific, Waltham, Massachusetts, USA), and aliquots were stored at − 80 °C for future analysis.

### Protein digestion

The filter aided sample preparation (FASP) method was applied in order to enzymatically digest protein [48]. A total of 100 μg of protein extraction was mixed with 120 μL of reducing buffer (pH = 8.0), containing 10 mM dithiothreitol (DTT, Sangon Biotech, Shanghai, China), 8 M urea, and 100 mM tetraethylammonium bromide (TEAB, Sigma-Aldrich, St. Louis, Missouri, USA), in a 10 K ultrafiltration tube. The solution was incubated at 60 °C for 1 h, and iodoacetamide (IAA, Sangon Biotech) was added in the dark at RT for 40 min to yield a final concentration of 50 mM. Afterward, solutions were centrifuged twice on the strainers at 12,000 rpm for 20 min at 4 °C, and liquid at the bottom was discarded. Then, 100 μL of 300 mM TEAB was added to every tube and centrifuged at 12,000 rpm for 20 min. The filter units were then transferred into new tubes with 100 μL of 300 mM TEAB. Subsequently, 3 μL of 1 μg/μL sequencing-grade trypsin was added to solutions and incubated at 37 °C for 12 h. Eventually, after digestion, peptides were collected and centrifuged at 12,000 rpm for 20 min. Finally, 50 μL of 200 mM TEAB was added and centrifuged once.

### iTRAQ labeling

After digestion, the lyophilized samples were resuspended in 100 μL of 200 mM TEAB, and one-fifth of each sample volume was transferred into a new collecting tube for labeling. Then, 200 μL of isopropanol was added to iTRAQ reagent vial (AB SCIEX, Toronto, Canada) and centrifuged twice. Afterward, 100 μL of iTRAQ label reagent was added to the samples and mixed. The mixture was incubated for 2 h. Ultimately, 200 µL of high-performance liquid chromatography (HPLC) water was added to the samples and incubated for 0.5 h for reaction termination. The labeled peptide solutions were freeze-dried in preparation for the next step.

### Reversed-Phase liquid chromatography (RPLC) analysis

Reversed-phase separation was conducted on an 1100 HPLC System (Agilent, Santa Clara, California, USA), and an Agilent Zorbax Extend RP column (5 μm, 150 mm × 2.1 mm) was applied. Mobile phases A (2% acetonitrile in HPLC water) and B (90% acetonitrile in HPLC water) were applied for RP gradient. The solvent gradient was set as follows: 0–8 min, 98% A; 8.00 min, 98–95% A; 8–48 min, 95–75% A; 48–60 min, 75–60% A; 60 min, 60–10% A; 60–70 min, 10% A; 70 min, 10–98% A; 70–75 min, 98% A. Tryptic peptides were separated at a velocity of 300 μL/min. Monitoring was at 210 nm and 280 nm. Samples were collected after 8 min and up to 60 min, and elution buffer was harvested every 1 min into centrifuge tubes. The separated peptides were lyophilized in a vacuum.

### Mass spectrometry

All analyses were conducted by a Q-Exactive mass spectrometer (Thermo Fisher Scientific). Samples were separated by a chromatographic column (PepMap C18, 100 Å, Thermo Fisher). The flow rate was set to 300 nL/min, and the gradient was 60 min (0–40 min, 5–30% B; 40–54 min, 30–50% B; 54–55 min, 50–100% B; 55–60 min, 100% B; mobile phase A = 0.1% FA in water and phase B = 80% ACN/0.1% FA in water).

Complete mass spectrometry (MS) scans were obtained in the mass range of 300–1600 m/z with a resolution of 35,000. The target value of automatic gain control (AGC) was 1e6. The 10 most intense peaks were fragmented using higher energy collisional dissociation (HCD). Normalized collision energy (NCE) was set to 30. MS/MS spectra were acquired with a resolution of 17,500, the AGC target was 2e5, and the maximum injection time was 50 ms. Q-Exact dynamic exclusion was set to 30 s and ran in positive mode.

### Proteomic data analysis

The resulting data were searched in the UniProt database, utilizing the *Rattus norvegicus* (Rat) taxonomy (https://www.uniprot.org/proteomes/UP000002494). Data were analyzed by Proteome Discoverer™ 2.2 (Thermo Fisher) software. The false discovery rate (FDR) of peptide search was controlled below 1%. At least one unique peptide was required to qualify a protein. Missed cleavage was set at 2. MS1 tolerant was set at 10 ppm and MS2 tolerant was 0.02 Da. The fixed modifications of iTRAQ 8-plex (N-term, K, Y), Carbamidomethyl (C) were specified.

Gene ontology enrichment (http://www.geneontology.org) and KEGG (http://www.genome.jp/kegg) pathway analyses of altered proteins were conducted by R based on the hypergeometric distribution.

### Parallel reaction monitoring

After extraction and digestion, proteins from eight samples were used for PRM (n = 4 per group). The immunoreactive trypsin (iRT) standard (Biognosys, Thermo Fisher) was dissolved to 10× and stored at 4 °C. The 10× iRT standard peptide mix was added to peptide samples before LC–MS injection. The ratio of iRT to samples was set at 1:10 v/v.

The samples were fractionated on an Agilent 1100 liquid chromatograph (pH = 10). Ultimately, six fractions were collected and run in data-dependent acquisition (DDA) mode. In short, the DDA raw files were searched on the database. In this database, the iRT peptide sequences with 11 entries were added with ProteomeDiscover (version 2.3). Fixed modifications were set to carbamidomethylation of cysteine, and variable modifications were set to oxidation of methionine and acetyl (protein N terminus). A maximum of two missed cleavages were permitted. The identifications were filtered to acquire 1% FDR at the peptide and protein levels. A list of peptides from DDA analysis was made for PRM validation (at least two peptides per protein). The flow rate was set to 300 nL/min. For PRM, precursors were targeted in a 1.2 m/z isolation window around the m/z of interest. Precursors were fragmented in HCD mode, and NCE energy was set to 32. MS/MS was performed at 30,000 resolution, and an AGC target of 5e5 spectra was manually verified using Skyline. The quantitative data were exported to an Excel spreadsheet, and the data were normalized to the total ion currant (TIC) of the MS runs.

### Levothyroxine treatment

Levothyroxine (MCE, HY-18341) is a synthetic form of T4 (referred to here as T4). T4 was infused 1 h after CCI. Rats were randomly divided into four groups: sham + saline, sham + T4, TBI + saline, and TBI + T4 (n = 15 per group). A total of 100 μg of T4 was dissolved in 10 mL of saline and used for i.p. injection. The sham + T4 and TBI + T4 groups received T4 at a concentration of 2.5 μg per 100 g of weight. The injection dose, treatment window, and method of delivery were set based on previous research [49–51].

### Modified neurological severity score

The modified neurological severity score (mNSS) was measured on days 1, 3, 5, and 7 post-CCI to evaluate the neurological function of the rats (n = 6 per group). The mNSS consists of motor, sensory, balance, and reflex tests. The highest scores of the four tests are 6, 2, 6, and 4, respectively. A higher score represents a more serious neurological deficit.

### Morris water maze

The Morris water maze (MWM) was used to test spatial learning ability and memory in rats (n = 6 per group). Briefly, from days 7 to 11 post-CCI, the rats were placed in a circular tank with water containing of Soft Gel Paste (AmeriColor, Placentia, California, USA) and trained to find an underwater platform. Four training sessions per day were conducted. On the first day, rats were placed from the first quadrant to the fourth quadrant, whereas on the second day, training started from the second quadrant to first quadrant, and on the third day, training started from the third quadrant to the second quadrant, and so on. Escape latency was recorded, and the mean time of four trainings on each day was calculated. On the test day (day 12), the platform was removed, and the rats were placed opposite from the platform quadrant. After a 120 s test, the escape latency, time spent in the platform quadrant, and number of times crossing the platform area were recorded by the automatic tracking system (Xmaze™, Xinruan Information Technology Co., Shanghai, China). Training and testing were performed by blinded researchers.

### Cresyl violet staining and lesion volume assessment

At 24 h post-CCI, rats (n = 3 per group) were anesthetized, and hearts were exposed by thoracotomy. Fifty milliliter of saline was infused to flush the blood, and 100 mL of 4% paraformaldehyde was injected for perfusion fixation. After perfusion, brains were removed and placed in paraformaldehyde at 4 °C for 24 h. After paraffin embedding, three 2-mm-thick coronal sections spanning across 6 mm to cover the whole damaged area were sliced and stained with cresyl violet (Solarbio, Beijing, China). Tissue volume loss was calculated by subtracting the ipsilateral volume (damaged hemisphere) from the contralateral volume (controlled hemisphere) [43]. The volumes of substructures were calculated by multiplying the area by section thickness. ImageJ software was applied to calculate the areas of substructures.

### Measurement of brain water content

At 24 h post-CCI, a wet-to-dry weight ratio was applied to reflect the degree of cerebral edema (n = 3 per group) [52]. After sacrifice, the brain parenchyma was extracted, and the cerebellum and olfactory bulb were discarded. The parenchyma was divided into two hemispheres: contralateral (control) and ipsilateral (damaged). Samples were weighed to acquire a wet weight immediately, then dried at 110 °C in a drying oven for 24 h to acquire a dry weight.

### Evaluation of blood–brain barrier (BBB) integrity

Rats were anesthetized 23 h post-CCI, and 2% Evans blue (EB) dye was injected through the caudal vein at a dose of 4 mL/kg and circulated for 1 h before sacrifice (n = 3 per group). The hearts were perfused with saline through the left ventricle until colorless perfusion was obtained. Afterwards, rats were sacrificed, and brains were removed and weighed. After homogenizing in 3 μL/mg formamide, samples were stored at RT for 48 h. Following centrifugation, the supernatant was collected, and the optical density was measured at 625 nm to detect the relative amount of EB.

### Statistical analysis

The Gaussian distribution for the quantitative proteomics data was analyzed using SPSS software (version 22.0, SPSS Chicago, Illinoi, USA) and Prism (version 8.0, San Diego, California, USA). Except proteomics and PRM data, other data were expressed as means ± standard deviation (SD). Student’s *t*-test was used for data analysis. A 2-tailed p value less than 0.05 was considered significant.

## Results

### iTRAQ analysis of differentially expressed proteins

**Fig. 1:**
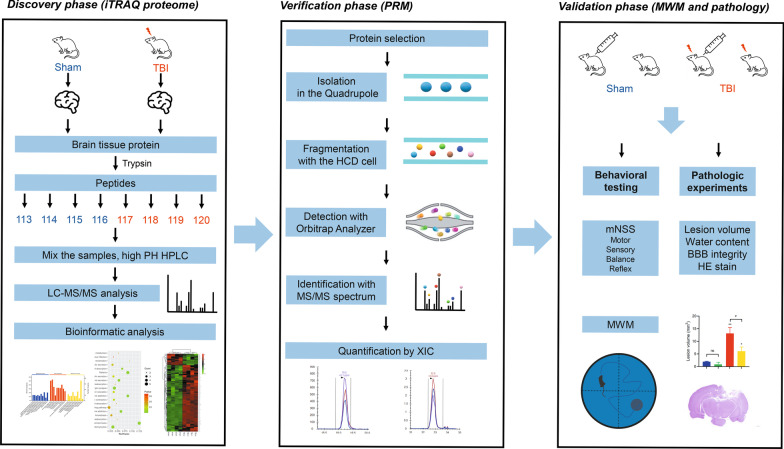
Systematic workflow of three phases in the study. In the discovery phase, 8-plex iTRAQ was conducted, and bioinformatic analysis was performed. In the verification phase, PRM was applied to verify the outcomes of quantitative proteomic analysis. In the validation phase, a protein of interest (transthyretin) was identified, and thyroxine was used to detect the effects. Behavioral outcomes and histopathology were observed, and conclusions were made

**Fig. 2:**
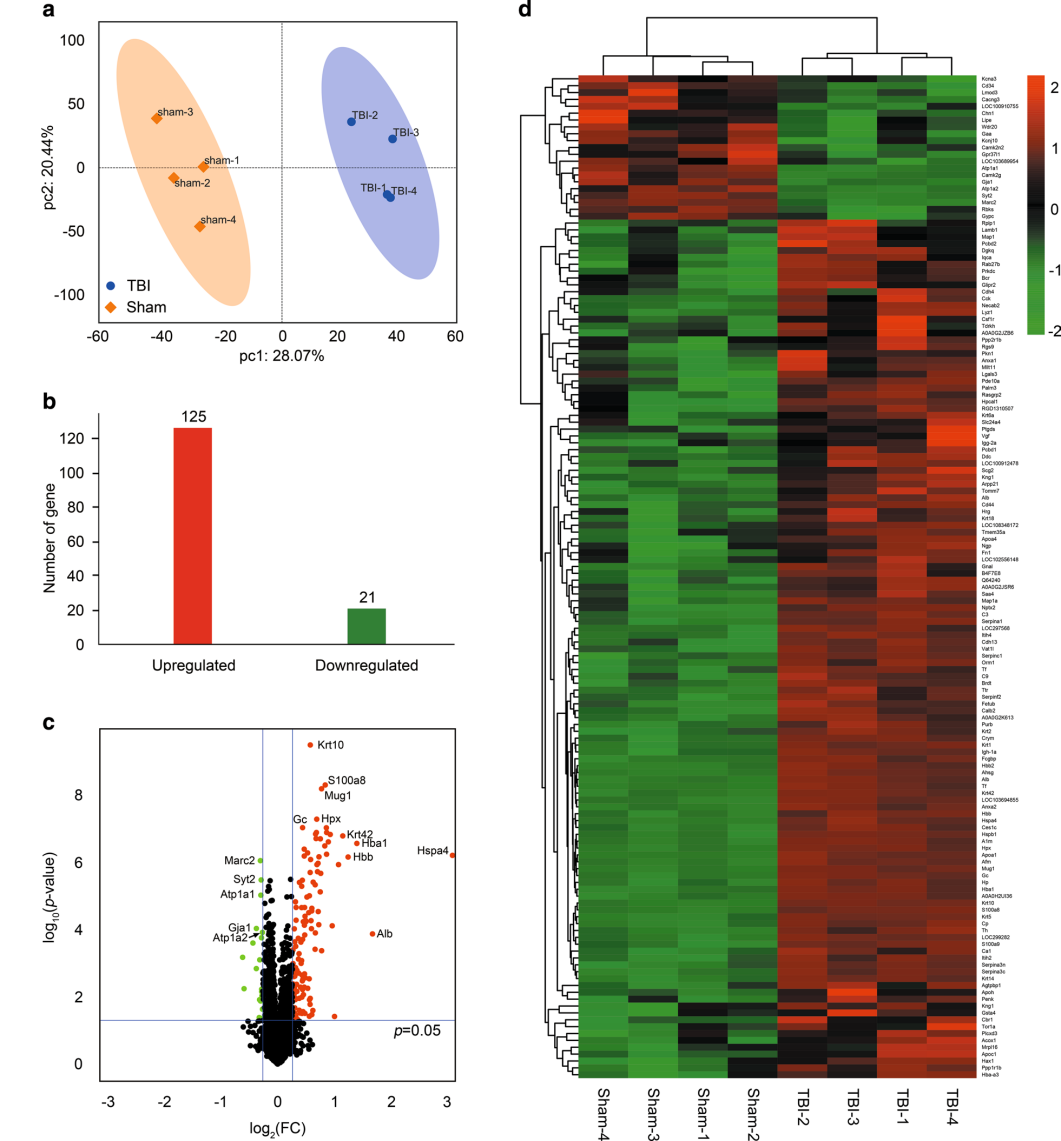
General proteomic outcomes. **a** Principal component analysis indicated a near complete separation of proteins in the TBI (blue) and sham (yellow) groups. **b** 125 proteins were upregulated, and 21 proteins were downregulated. **c** Volcano plot showed differentially expressed proteins. The red and green dots indicate significantly upregulated and downregulated proteins, respectively. **d** Hierarchical cluster analysis. Red indicates upregulation, and green indicates downregulation. The dendrograms represent the classification of proteins. The number in the color scale indicates z-score

Eight brain cortical samples were isolated from two groups and analyzed using iTRAQ label with LC–MS/MS (Fig. 1). Principle component analysis (PCA) was performed to compare proteomes between the TBI and sham groups, and it was observed that the groups of samples were well-clustered (Fig. 2a). Based on the discovery analysis, a total of 3937 proteins were identified and quantified, of which 125 proteins were upregulated and 21 proteins were downregulated significantly (fold change > 1.20 and < 5/6, *p* < 0.05 as threshold; Fig. 2b; Table 1). The top five proteins based on the fold change included Hspa4, Alb, Hba1, Hbb, and Krt42. The top five proteins based on the *p*-value included Krt10, S100a8, Mug1, Hpx, and Gc (Fig. 2c). Hierarchical clustering analysis (heat map) was generated to show differential protein patterns among samples and groups. As shown in Fig. 2d, two groups showed disparate protein change patterns, and most of the samples within one group were upregulated or downregulated consistently, which indicated good reproducibility. Additional file 1: Table S1 shows raw data of all identified proteins and differentially expressed proteins by iTRAQ.

**Table 1:** Differentially expressed proteins after TBI by iTRAQ

Accession	Description	Coverage (%)	MW (kDa)	Average		p-value	FC
				Sham	TBI		
D3ZVU4	Ribokinase (Rbks)	11	34	121.2	78.8	0.00070	0.65
P30337	N-chimaerin (Chn1)	4	38.2	120.4	79.7	0.0057	0.66
B1PLB1	CD34 antigen (Cd34)	11	41.2	115.1	84.9	0.00030	0.74
Q6XFR6	Glycophorin-C (Gypc)	27	10.4	113.0	87.0	0.0014	0.77
P08050	Gap junction alpha-1 protein (Gja1)	17	43	113.0	87.0	0.00010	0.77
P15384	Potassium voltage-gated channel subfamily A member 3 (Kcna3)	8	58.4	111.3	88.7	0.042	0.80
P49655	ATP-sensitive inward rectifier potassium channel 10 (Kcnj10)	4	42.5	111.1	88.9	0.012	0.80
Q6P7A9	Lysosomal alpha-glucosidase (Gaa)	4	106.1	110.9	89.0	0.00080	0.80
D3Z900	Mitochondrial amidoxime reducing component 2 (Marc2)	16	38.2	110.6	89.4	< 0.0001	0.81
D4A871	Leiomodin 3 (Lmod3)	2	66	110.5	89.5	0.013	0.81
P06685	Sodium/potassium-transporting ATPase subunit alpha-1 (Atp1a1)	46	113	110.3	89.7	< 0.0001	0.81
D3ZSW9	RUN and FYVE domain-containing 2 (LOC100910755)	2	69.9	110.3	89.8	0.013	0.81
P29101	Synaptotagmin-2 (Syt2)	31	47.2	110.1	89.9	< 0.0001	0.82
P11730	Calcium/calmodulin-dependent protein kinase type II subunit gamma (Camk2g)	28	59	109.9	90.1	0.00020	0.82
B4F7C1	G protein-coupled receptor 37-like 1, isoform CRA_a (Gpr37l1)	2	52.8	109.8	90.2	0.0060	0.82
D4A617	Ectonucleoside triphosphate diphosphohydrolase 1 (LOC103689954)	3	52	109.8	90.2	0.0071	0.82
Q8VHX0	Voltage-dependent calcium channel gamma-3 subunit (Cacng3)	10	35.5	109.6	90.4	0.0093	0.82
P06686	Sodium/potassium-transporting ATPase subunit alpha-2 (Atp1a2)	45	112.1	109.4	90.7	0.00010	0.83
A0A0G2K2B7	WD repeat domain 20 (Wdr20)	4	64.6	109.3	90.7	0.023	0.83
G3V8R5	Hormone-sensitive lipase (Lipe)	1	116.8	109.3	90.8	0.044	0.83
Q9Z2N6	Calcium/calmodulin-dependent protein kinase II inhibitor 2 (Camk2n2)	16	8.6	109.1	90.9	0.0095	0.83
P20760	Ig gamma-2A chain C region (Igg-2a)	16	35.2	90.8	109.3	0.022	1.20
Q9QYJ6	cAMP and cAMP-inhibited cGMP 3′,5′-cyclic phosphodiesterase 10A (Pde10a)	14	90.1	90.7	109.4	0.00040	1.21
D3ZMR1	Translocase of outer mitochondrial membrane 7 (Tomm7)	11	6.2	90.6	109.4	0.0010	1.21
P14173	Aromatic-l-amino-acid decarboxylase (Ddc)	11	54	90.6	109.4	0.0042	1.21
Q99P74	Ras-related protein Rab-27B (Rab27b)	5	24.6	90.6	109.4	0.0062	1.21
M0RDW3	*N*-acetyltransferase 8 (GCN5-related) family member 4 (LOC102556148)	3	25.6	90.4	109.6	0.0079	1.21
D3ZM03	Vacuolar ATPase assembly integral membrane protein VMA21 (LOC100912478)	12	11.4	90.3	109.8	0.0044	1.22
D3ZCC3	Slc24a4 protein (Slc24a4)	4	62.8	90.3	109.8	0.045	1.22
P14942	Glutathione S-transferase alpha-4 (Gsta4)	8	25.5	90.1	110.0	0.043	1.22
R9PXW6	RAS guanyl releasing protein 2, isoform CRA_b (Rasgrp2)	5	69.3	90.0	110.0	0.0025	1.22
F1LXF1	BCR, RhoGEF and GTPase-activating protein (Bcr)	6	120	90.0	110.0	0.0088	1.22
Q4QQT4	Serine/threonine-protein phosphatase 2A 65 kDa regulatory subunit A beta isoform (Ppp2r1b)	11	66	89.8	110.2	0.046	1.23
Q5FVI0	Arpp-21 protein (Arpp21)	34	9.6	89.8	110.2	0.00010	1.23
Q6JAM9	Transmembrane protein 35A (Tmem35a)	5	18.5	89.8	110.3	0.0054	1.23
D3ZEY4	Diacylglycerol kinase (Dgkq)	3	102.5	89.7	110.3	0.0078	1.23
A0A0G2JXN6	Galectin (Lgals3)	4	27.6	89.7	110.3	0.031	1.23
D4A1H2	Phosphatidylinositol-specific phospholipase C, X domain-containing 3 (Plcxd3)	8	36.3	89.5	110.5	0.021	1.23
F1LQY6	N-terminal EF-hand calcium-binding protein 2 (Necab2)	16	43.5	89.5	110.5	0.00020	1.23
F1LST1	Fibronectin (Fn1)	4	262.6	89.5	110.5	0.0024	1.24
D4A1J3	Paralemmin 3 (Palm3)	7	78.5	89.3	110.7	0.0020	1.24
Q5M971	Protein AF1q (Mllt11)	8	10	89.2	110.8	0.012	1.24
Q5M7T5	Serine (Or cysteine) peptidase inhibitor, clade C (Antithrombin), member 1 (Serpinc1)	16	52.2	89.1	110.9	< 0.0001	1.24
A0A0G2K896	Similar to RIKEN cDNA 1300017J02 (RGD1310507)	5	76.7	89.1	111.0	0.0014	1.25
P34926	Microtubule-associated protein 1A OS = Rattus norvegicus OX = 10,116 GN = Map1a PE = 1 SV = 1	32	299.3	89.0	111.1	0.00030	1.25
A0A0G2K728	Cadherin-4 (Cdh4)	3	94.4	89.0	111.1	0.035	1.25
P49805	Regulator of G-protein signaling 9 (Rgs9)	2	77.1	88.9	111.1	0.041	1.25
P22057	Prostaglandin-H2 d-isomerase (Ptgds)	9	21.3	88.8	111.2	0.011	1.25
Q8R490	Cadherin 13 (Cdh13)	13	78	88.7	111.3	0.00010	1.25
A0A0G2K613	Uncharacterized protein	22	19.8	88.7	111.3	< 0.0001	1.25
F1M7Z2	IQ and AAA domain-containing protein 1 (Iqca)	1	99.3	88.5	111.6	0.0043	1.26
Q7TSE9	HCLS1-associated protein X-1 (Hax1)	9	31.4	88.3	111.8	0.0070	1.27
Q6J4I0	Protein phosphatase 1 regulatory subunit 1B (Ppp1r1b)	32	22.9	88.3	111.7	0.0016	1.27
P19944	60S acidic ribosomal protein P1 (Rplp1)	57	11.5	87.9	112.1	0.047	1.28
A0A0G2KAY3	Kininogen-1 (Kng1)	12	44.2	87.4	112.6	0.00020	1.29
A0A0G2JSR6	Guanine nucleotide-binding protein subunit gamma (Gng7)	54	7.7	87.3	112.7	0.00080	1.29
P61459	Pterin-4-alpha-carbinolamine dehydratase (Pcbd1)	7	12	87.2	112.8	0.0020	1.29
B4F7E8	Niban-like protein 1 (Fam129b)	3	84.7	87.0	113.1	0.00050	1.30
F1M5V2	GLI pathogenesis-related 2 (Glipr2)	8	19	86.9	113.2	0.025	1.30
P19939	Apolipoprotein C-I (Apoc1)	10	9.9	86.8	113.2	0.011	1.30
Q5EBC0	Inter alpha-trypsin inhibitor, heavy chain 4 (Itih4)	9	103.7	86.7	113.3	< 0.0001	1.31
G3V8T7	RCG51933, isoform CRA_a (Tdrkh)	4	62	86.3	113.8	0.031	1.32
P47727	Carbonyl reductase [NADPH] 1 (Cbr1)	39	30.6	86.1	113.9	0.0052	1.32
A0A0G2JVX7	Apolipoprotein A-IV (Apoa4)	23	44.3	85.8	114.2	0.00020	1.33
P04094	Proenkephalin-A (Penk)	12	30.9	85.7	114.3	0.0010	1.33
A0A0G2JZB6	Uncharacterized protein	12	15.8	85.6	114.4	0.029	1.34
P02764	Alpha-1-acid glycoprotein (Orm1)	11	23.6	85.4	114.6	< 0.0001	1.34
P62749	Hippocalcin-like protein 1 (Hpcal1)	26	22.3	85.4	114.6	0.00080	1.34
P04177	Tyrosine 3-monooxygenase (Th)	28	55.9	85.3	114.7	< 0.0001	1.34
D3ZTN0	Protein kinase, DNA-activated, catalytic polypeptide (Prkdc)	0	471.8	85.2	114.8	0.010	1.35
Q00495	Macrophage colony-stimulating factor 1 receptor (Csf1r)	1	109.2	85.2	114.8	0.031	1.35
G3V7X2	Scg2 protein (Scg2)	4	66.6	85.1	114.9	0.0023	1.35
Q9QYU4	Ketimine reductase mu-crystallin (Crym)	39	33.5	85.0	115.1	< 0.0001	1.35
P07150	Annexin A1 (Anxa1)	17	38.8	84.9	115.1	0.0034	1.35
A0A0G2JZV3	ATP/GTP-binding protein 1 (Agtpbp1)	3	136.5	84.8	115.2	0.0061	1.36
Q68FY4	Group specific component (Gc)	28	53.5	84.8	115.2	< 0.0001	1.36
Q64240	Protein AMBP (Ambp)	8	38.8	84.8	115.2	0.00020	1.36
A0A0G2K3G0	Histidine-rich glycoprotein (Hrg)	4	60.3	84.7	115.3	0.0031	1.36
P08934	Kininogen-1 (Kng1)	5	70.9	84.6	115.4	0.028	1.37
Q9QX79	Fetuin-B (Fetub)	15	41.5	84.4	115.6	0.00010	1.37
F1M609	Acyl-coenzyme A oxidase (Acox1)	3	66	84.3	115.7	0.0086	1.37
A0A0G2K9I6	Ceruloplasmin (Cp)	10	123.6	83.8	116.2	< 0.0001	1.39
D3ZY96	Neutrophilic granule protein (Ngp)	20	19.4	83.8	116.2	0.0052	1.39
Q07936	Annexin A2 (Anxa2)	12	38.7	83.8	116.2	< 0.0001	1.39
D3ZGF1	CD44 antigen (Cd44)	7	52.8	83.7	116.3	0.0001	1.39
G3V6X7	ProSAAS (LOC108348172)	13	27.5	83.7	116.4	0.0021	1.39
M0R3N4	Vesicle amine transport 1-like (Vat1l)	22	41.6	83.6	116.4	< 0.0001	1.39
Q5M878	Serum amyloid A protein (Saa4)	15	15	83.5	116.5	0.00010	1.39
Q5M818	39S ribosomal protein L16, mitochondrial (Mrpl16)	6	28.9	83.4	116.6	0.031	1.40
A0A0G2JWX4	Keratin, type II cytoskeletal 2 epidermal (Krt2)	6	69.1	83.4	116.6	< 0.0001	1.40
Q63433	Serine/threonine-protein kinase N1 (Pkn1)	1	104.4	83.2	116.8	0.0027	1.40
A0A0G2K526	Guanine nucleotide-binding protein G(olf) subunit alpha (Gnal)	9	51.3	83.0	117.0	0.00010	1.41
P97738	Neuronal pentraxin-2 (Nptx2)	18	47.4	82.8	117.2	0.00010	1.42
P20761	Ig gamma-2B chain C region (Igh-1a)	7	36.5	82.2	117.8	< 0.0001	1.43
A0A0G2JYK0	Serine protease inhibitor A3N (LOC299282)	27	67.9	81.8	118.2	< 0.0001	1.44
Q63910	Alpha globin (Hba-a3)	15	15.5	81.8	118.3	0.0056	1.45
P47728	Calretinin (Calb2)	15	31.4	81.2	118.9	< 0.0001	1.46
P01355	Cholecystokinin (Cck)	9	12.8	80.9	119.1	0.0029	1.47
D3ZQN7	Laminin subunit beta 1 (Lamb1)	1	197.3	80.8	119.2	0.038	1.48
D4AA52	Murinoglobulin-1 (LOC297568)	13	163.6	80.7	119.3	< 0.0001	1.48
P01048	T-kininogen 1 (Map1)	6	47.7	80.7	119.3	0.012	1.48
Q4FZU2	Keratin, type II cytoskeletal 6A (Krt6a)	7	59.2	80.4	119.7	0.017	1.49
P06866	Haptoglobin (Hp)	19	38.5	80.3	119.7	< 0.0001	1.49
Q5I0M1	Apolipoprotein H (Apoh)	11	38.4	80.3	119.8	0.011	1.49
Q6IFW6	Keratin, type I cytoskeletal 10 (Krt10)	13	56.5	80.2	119.8	< 0.0001	1.49
A0A0G2QC06	Serotransferrin (Tf)	24	107.3	80.1	120.0	< 0.0001	1.50
P02767	Transthyretin (Ttr)	20	15.7	79.8	120.2	0.00010	1.51
F7FHF3	Serpin family F member 2 (Serpinf2)	5	62.3	79.6	120.5	0.00010	1.51
D3ZFH5	Inter-alpha-trypsin inhibitor heavy chain 2 (Itih2)	4	92.3	79.4	120.6	< 0.0001	1.52
Q68G38	Torsin-1A (Tor1a)	3	37.9	79.3	120.7	0.034	1.52
A0A0G2JUX5	Transcriptional activator protein Pur-beta (Purb)	24	33.5	79.3	120.7	< 0.0001	1.52
P20156	Neurosecretory protein VGF (Vgf)	21	68.1	78.7	121.3	0.026	1.54
M0RBF1	Complement C3 (C3)	21	186.2	78.6	121.5	< 0.0001	1.55
P00697	Lysozyme C-1 (Lyz1)	16	16.7	77.4	122.6	0.00040	1.58
A0A0G2JZ73	Alpha-1-antiproteinase (Serpina1)	39	46.6	77.4	122.6	< 0.0001	1.59
Q62930	Complement component C9 (C9)	2	62.2	77.3	122.8	0.00010	1.59
Q6P6Q2	Keratin, type II cytoskeletal 5 (Krt5)	15	61.8	77.1	122.9	< 0.0001	1.59
G3V9R9	Afamin (Afm)	15	69.2	76.9	123.2	< 0.0001	1.60
P24090	Alpha-2-HS-glycoprotein (Ahsg)	26	38	76.6	123.4	< 0.0001	1.61
P20059	Hemopexin (Hpx)	23	51.3	76.5	123.6	< 0.0001	1.62
A0A0G2JSK1	RCG20603 (Serpina3c)	28	46.4	76.3	123.7	< 0.0001	1.62
A0A0H2UHJ1	Protein S100-A9 (S100a9)	11	15.2	76.0	124.0	< 0.0001	1.63
Q6IMF3	Keratin, type II cytoskeletal 1 (Krt1)	8	64.8	75.7	124.3	< 0.0001	1.64
F7F8G2	Bromodomain testis-specific protein (Brdt)	1	106.7	75.6	124.4	< 0.0001	1.65
P12346	Serotransferrin (Tf)	31	76.3	75.3	124.7	0.00010	1.66
D3ZJF8	Fc fragment of IgG-binding protein (Fcgbp)	0	275	75.2	124.9	< 0.0001	1.66
A0A0H2UHI5	Serine protease inhibitor A3N (Serpina3n)	24	45.5	74.4	125.6	< 0.0001	1.69
Q63041	Alpha-1-macroglobulin (A1m)	10	167	74.4	125.7	< 0.0001	1.69
P10959	Carboxylesterase 1C (Ces1c)	11	60.1	74.2	125.9	< 0.0001	1.70
Q03626	Murinoglobulin-1 (Mug1)	13	165.2	73.7	126.3	< 0.0001	1.71
Q5BJY9	Keratin, type I cytoskeletal 18 (Krt18)	6	47.7	73.6	126.4	0.00040	1.72
A0A0H2UI36	Uncharacterized protein	14	17	71.9	128.2	< 0.0001	1.78
P50115	Protein S100-A8 (S100a8)	22	10.2	71.6	128.4	< 0.0001	1.79
P42930	Heat shock protein beta-1 (Hspb1)	41	22.9	71.0	129.0	< 0.0001	1.82
Q6IFV1	Keratin, type I cytoskeletal 14 (Krt14)	15	52.7	70.8	129.2	< 0.0001	1.82
P04639	Apolipoprotein A-I (Apoa1)	41	30	70.8	129.2	< 0.0001	1.83
P02770	Serum albumin (Alb)	64	68.7	69.9	130.2	< 0.0001	1.86
Q62669	Globin a1 (LOC103694855)	46	16	68.8	131.2	< 0.0001	1.91
B0BNN3	Carbonic anhydrase 1 (Ca1)	8	28.3	67.7	132.3	0.00010	1.95
M0RCX0	Pterin-4 alpha-carbinolamine dehydratase 2 (Pcbd2)	22	14.1	66.4	133.6	0.039	2.01
P02091	Hemoglobin subunit beta-1 (Hbb)	82	16	64.3	135.7	< 0.0001	2.11
Q6IFU7	Keratin, type I cytoskeletal 42 (Krt42)	13	50.2	62.0	138.0	< 0.0001	2.22
P11517	Hemoglobin subunit beta-2 (Hbb2)	78	16	59.3	140.8	< 0.0001	2.38
P01946	Hemoglobin subunit alpha-1/2 (Hba1)	63	15.3	54.9	145.1	< 0.0001	2.64
A0A0G2JSH5	Serum albumin (Alb)	65	68.7	47.6	152.4	0.00010	3.21
F1LRV4	Heat shock 70 kDa protein 4 (Hspa4)	41	94	20.9	179.1	< 0.0001	8.56

*MW* molecular weight, *TBI* traumatic brain injury, *FC* fold change

### Gene ontology analysis

**Fig. 3:**
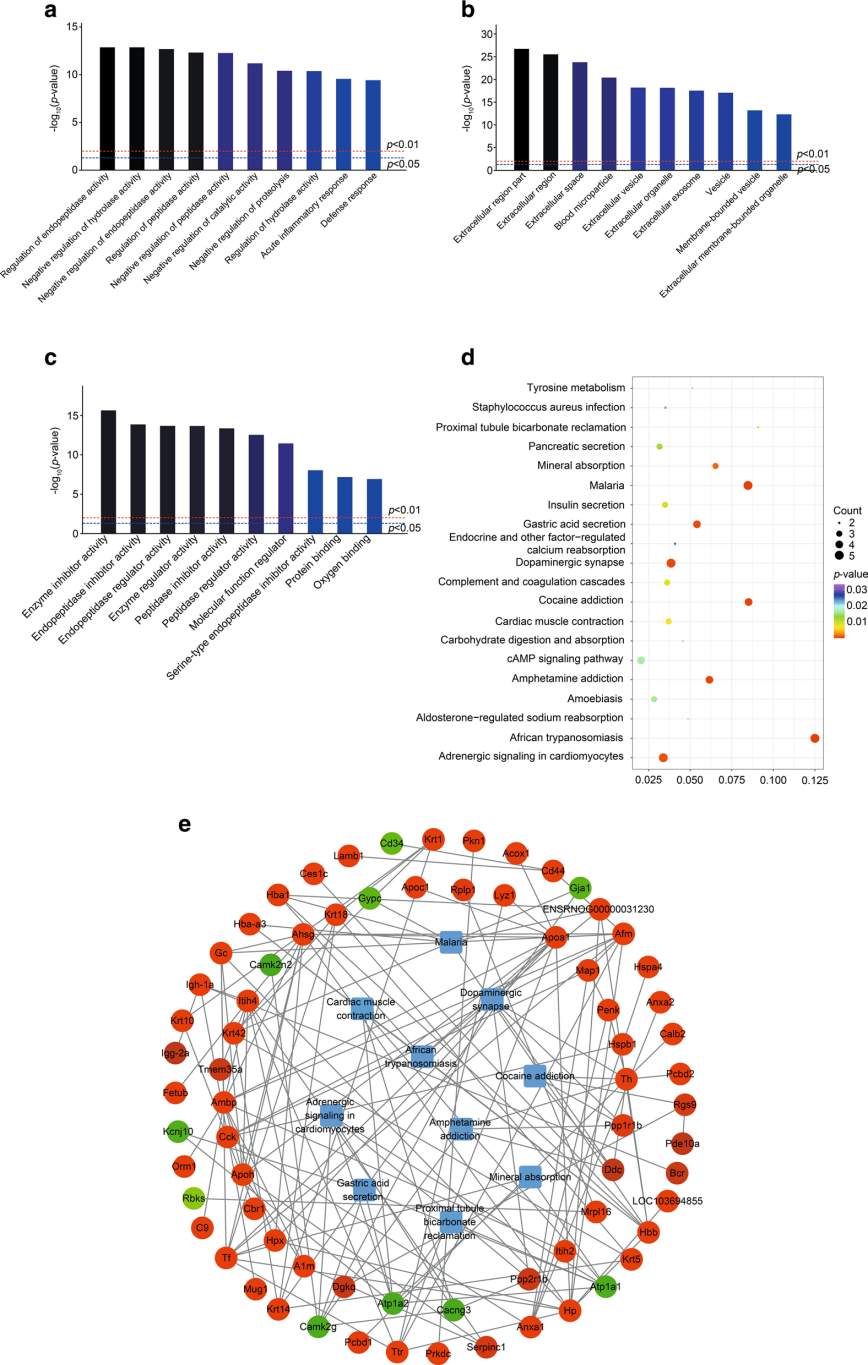
Bioinformatic analysis for differentially expressed proteins in the TBI and sham rats. **a** Biological process (BP). **b** cell components (CC). **c** molecular function (MF). Darker colors indicate higher statistical significancy. **d** Bubble chart shows KEGG analysis of differential genes. The horizontal axis represents rich factor (ratio of the sum of differential genes enriched in a pathway to the number of genes annotated by the pathway). Bubble size indicates the number of proteins included in each pathway, and different colors indicate different *p*-values. **e** Protein–protein interaction (red = upregulated protein; green = downregulated protein). Rounded rectangles represent KEGG pathways. Lines show interactions between multiple proteins or between proteins and pathways

By GO analysis, the biological process (BP), cellular component (CC), and molecular function (MF) of genes in different proteins were annotated. As Fig. 3a shows, the most overrepresented biological processes were related to regulation of endopeptidase, hydrolase, and peptidase, as well as negative regulation of catalytic activity and proteolysis. In CC annotation, the highly enriched terms included the extracellular region, extracellular space, blood microparticle, extracellular vesicle, extracellular organelles, and extracellular exosome (Fig. 3b). From this, it can be inferred that most differentially expressed proteins were extracellular, including Ttr. The molecular function annotation was consistent with BP, including inhibition of enzymes such as endopeptidase and peptidase. Some proteins were also related to protein binding (i.e., Ttr and Camk2g) and oxygen binding (i.e., Alb, Hbb, and Hba1; Fig. 3c).

### KEGG analysis

To reveal the pathways involved in proteins that were differentially expressed between TBI and sham groups, KEGG pathway analysis was employed. As shown in Fig. 3d, the most significantly enriched pathway, and with the highest rich factor, was African trypanosomiasis. Hemoglobin-related proteins such as Hbb, Hba1, Hba-a3, and ApoA1 were enriched in this pathway. The malaria pathway is similar to African trypanosomiasis, as proteins associated with this pathway also all originated from blood. Cocaine addiction, amphetamine addiction, and dopaminergic synapses were also significant pathways and shared altered proteins such as protein phosphatase 1 regulatory subunit 1B (Ppp1r1b), tyrosine hydroxylase (Th), and DOPA decarboxylase (Ddc), which are all associated with dopamine synthesis, except for calcium/calmodulin-dependent protein kinase type II subunit gamma (Camk2g), which is thought to be a mediator of memory by playing an important role in memory destabilization [53]. Other pathways with top 20 enrichment score values included gastric acid secretion, adrenergic signaling in cardiomyocytes, mineral absorption, proximal tubule bicarbonate reclamation, cardiac muscle contraction, complement and coagulation cascades, insulin secretion, pancreatic secretion, amoebiasis, tyrosine metabolism, cAMP signaling pathway, aldosterone-regulated sodium reabsorption, carbohydrate digestion and absorption, endocrine and other factor-regulated calcium reabsorption, and *Staphylococcus aureus* infection.

### Protein–protein interaction analysis

In order to further comprehend the interactions among the altered proteins, bioinformatics analysis (in this case, PPI) was performed. According to Fig. 3e, upregulated proteins (Ppp1r1b, Th, and Ddc) and one downregulated protein (Camk2g) were more relevant to cocaine addiction and amphetamine addiction pathways. Interestingly, proteins associated with myocardium-related pathways and gastric acid secretion pathways were all downregulated (i.e., Atp1a2, Atp1a1, Camk2g, Kcnj10, and Cacng3).

As Fig. 3e shows, the following proteins were identified: Ttr interacting with Tf (serotransferrin), ApoA1, Hp (haptoglobin), Itih4 (inter-alpha-trypsin inhibitor, heavy chain 4), Afm (afamin), and lysozyme C-1. Ambp (alpha-1-microglobulin/bikunin precursor) interacted with A1m (alpha-1-microglobulin), Hpx (hemopexin), Gc (GC vitamin D binding protein), Ahsg (alpha 2-HS glycoprotein), ApoA1, Apoh, Itih4, Itih2, Afm, C9, and Orm1 (orosomucoid 1). Th interacted with Ddc, Pcbd1 (pterin-4 alpha-carbinolamine dehydratase), Pcbd2, Calb2 (calbindin 2), Penk (proenkephalin), and Hbb. Hspb1 (heat shock protein b1) interacted with Hspa4, Anxa1 (annexin A1), and Cck (cholecystokinin).

### Validation of differentially expressed proteins by PRM

**Fig. 4:**
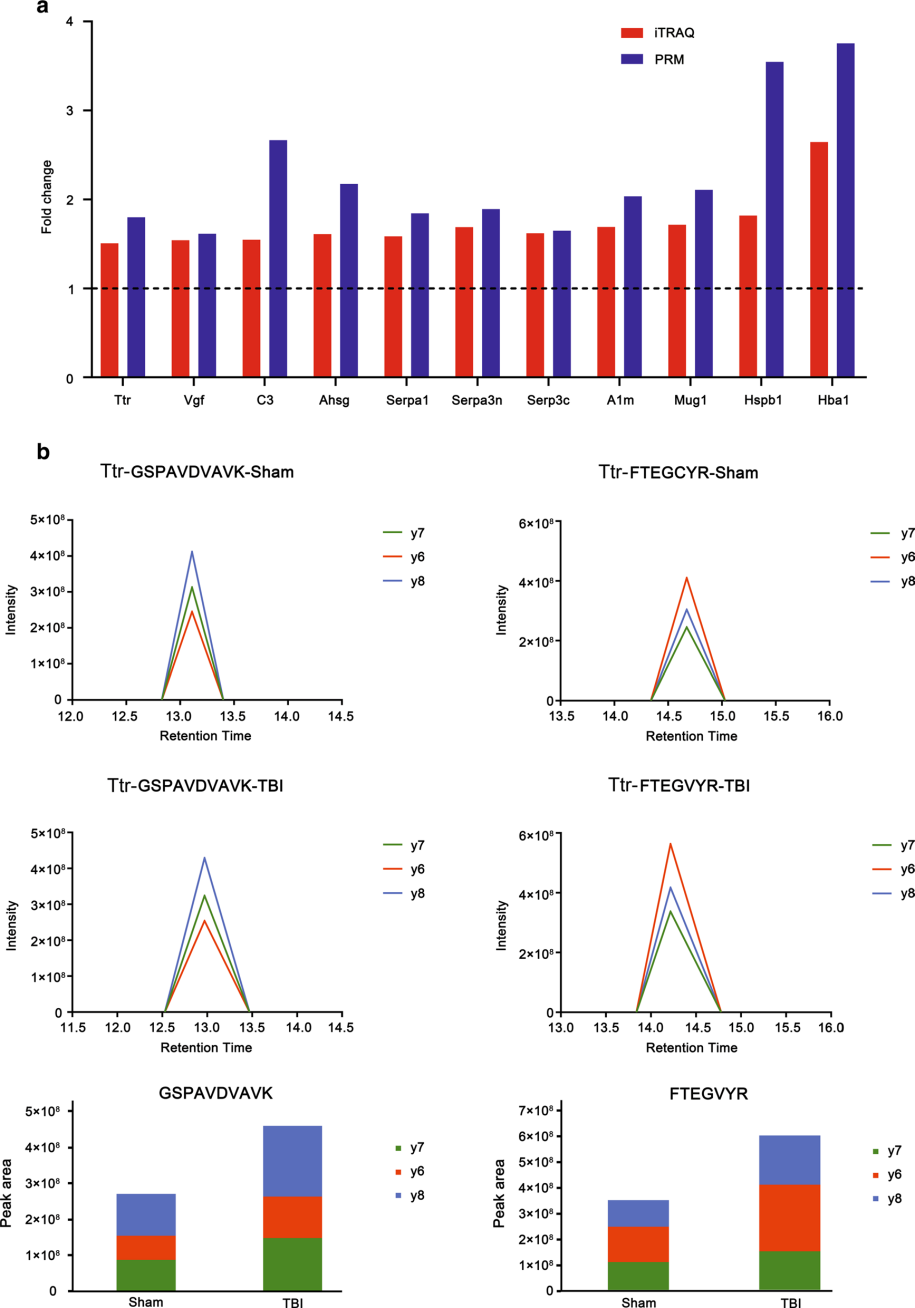
Parallel reaction monitoring outcomes. **a** Comparison of fold changes of some proteins between iTRAQ and PRM methods. A fold change > 1 indicates upregulation. **b** Retention time and intensity of two peptides of Ttr fitted from four samples each group (upper). Peak areas of two peptides were displayed as a histogram below

A PRM assay was developed to verify the abundance alterations of 23 upregulated proteins formerly identified by quantitative iTRAQ analysis (Fig. 1). As illustrated in Table 2, among the 23 proteins, seven were inconsistent with previous outcomes yielded by quantitative analysis. Among these, F1LRV4 (Hspa4) and Q6IFU7 (Krt42) were not significantly changed, while Q6IFV1, Q6IMF3, Q6P6Q2, D3ZJF8 and G3V9R9 (Krt14, Krt1, Krt5, Fcgbp and Afamin) were downregulated. The other 16 proteins were consistent with previous iTRAQ analysis outcomes, 11 of which were displayed in Fig. 4a. Additional file 2: Table S2 showed detailed quantitation of all peptides and proteins. The intensities and peak areas of two peptides in TTR were shown in Fig. 4b. The accurate intensities and peak areas of two peptides in each sample were shown in Additional file 3: Figure S1 and Additional file 4: Figure S2. The intensity of TTR ascended significantly after CCI, in agreement with the iTRAQ analysis.

**Table 2:** Verification of differentially expressed proteins after TBI by PRM

Accession	Gene name	Average total normalized area		Fold change	p-value
		Sham	TBI		
A0A0G2JSK1	Serpina3c	587,193.8154	967,199.9746	1.65	0.0010
A0A0G2JZ73	Serpina1	358,594,583.9	661,025,493	1.84	< 0.0001
A0A0H2UHI5	Serpina3n	15,925,972.06	30,105,445.75	1.89	< 0.0001
D3ZJF8	Fcgbp	492,977,070	364,252,286	0.74	0.00020
F1LRV4	Hspa4	315,888,493.8	319,391,993	1.01	0.48
G3V9R9	Afm	9,171,981.438	6,262,567.344	0.68	0.058
M0RBF1	C3	33,341,743.73	88,878,077.44	2.66	< 0.0001
P01946	Hba1	859,325,340.5	3,225,272,654	3.75	< 0.0001
P02091	Hbb	2,007,838,409	6,847,982,715	3.41	< 0.0001
P02767	Ttr	152,664,429.5	274,579,978.7	1.80	< 0.0001
P02770	Alb	1,334,438,359	1,462,217,836	1.10	0.099
P11517	Hbb2	1,987,823,049	6,870,701,039	3.46	< 0.0001
P20059	Hpx	81,400,524.38	143,040,114.8	1.76	< 0.0001
P20156	Vgf	17,525,134.63	28,274,163.63	1.61	< 0.0001
P24090	Ahsg	11,552,915.97	25,108,376.28	2.17	< 0.0001
P42930	Hspb1	5,132,833.438	18,194,532.5	3.54	< 0.0001
Q03626	Mug1	70,921,905.59	149,480,671.3	2.11	< 0.0001
Q62669	LOC103694855	487,524,419.4	1,385,746,698	2.84	< 0.0001
Q63041	A1m	9,261,727.438	18,843,111.94	2.03	< 0.0001
Q6IFU7	Krt42	40,789,820	35,523,088.63	0.87	0.31
Q6IFV1	Krt14	927,194,854	778,350,430.6	0.84	0.00030
Q6IMF3	Krt1	1,326,149,813	947,186,885.5	0.71	< 0.0001
Q6P6Q2	Krt5	623,317,271.3	427,289,138.7	0.69	0.0095

*TBI* traumatic brain injury; *PRM* parallel reaction monitoring

### Histopathology after T4 treatment

**Fig. 5:**
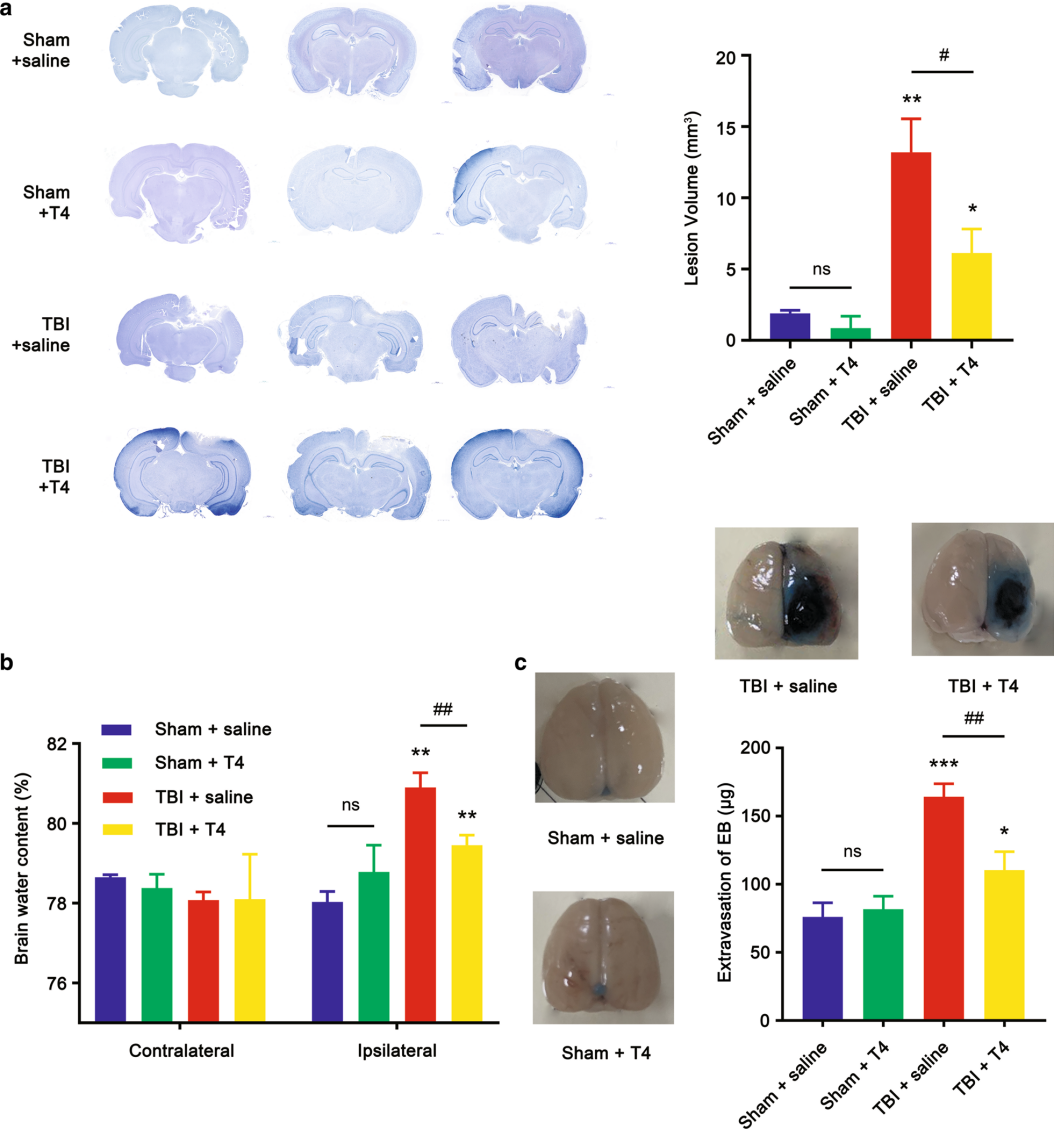
Effects of T4 on histopathology post-TBI. **a** Brain sections stained with cresyl violet were shown on the left. Lesion volume was calculated from the sections (n = 3 per group). **b** Brain water content of traumatized hemisphere increased 1 day post-TBI, whereas T4 alleviates edema (n = 3 per group). **c** BBB integrity was reflected by detecting the extravasation of EB dye (n = 3 per group). Brain samples from four groups were shown (*p < 0.05, **p < 0.01, and ***p < 0.001 vs. sham + saline; ^#^*p* < 0.05 and ^##^*p* < 0.01 vs. TBI + saline; ns: not significant). Error bars represent standard deviation (SD)

To evaluate the effects of T4 on alleviation of TBI-induced cerebrovascular histopathology, brain water content and BBB integrity were examined (Fig. 1). First, brain lesion volumes in the four groups (sham + saline, sham + T4, TBI + saline, and TBI + T4) were examined, and it was found that T4 significantly reduced lesion size in TBI rats, as reflected in brain sections shown in Fig. 5a. Lesion volume in the TBI + saline group was significantly higher compared with the sham + saline group (1.90 ± 0.21 vs. 13.20 ± 2.34 mm^3^, *p* = 0.0011). When comparing lesion volume in the TBI + T4 group, the difference was also significant (13.20 ± 2.34 vs. 6.12 ± 1.69, *p* = 0.013).

Brain water content was detected using a wet-to-dry ratio. According to Fig. 5b, there was no change in water content between two sham groups (exposed or not exposed to T4 [78.03 ± 0.26% vs. 78.78 ± 0.67%, *p* = 0.14]), while water content in the ipsilateral hemisphere significantly increased after CCI without exposure to T4 (78.03 ± 0.26% vs*.* 80.90 ± 0.37%, *p* = 0.0085). After CCI rats were exposed to T4, the water content significantly decreased (79.46 ± 0.25%, *p* = 0.0049), but it was still significantly higher than the sham + saline group (*p* = 0.0024). This indicated that the addition of T4 may improve brain edema. The extravasation of EB dye was examined and is shown in Fig. 5c. After 24 h post-CCI, the extravasation significantly increased in the TBI + saline group (76.16 ± 10.19 μg/g vs. 164.30 ± 9.44 μg/g, *p* = 0.0004). Extravasation of the TBI + T4 group was also significantly lower than the TBI + saline group (110.30 ± 13.62 μg/g, *p* = 0.0049). However, it was also higher than the sham + saline group (*p* = 0.025). This indicated improved BBB integrity after exposure to T4. Brain samples also confirmed these results. In general, the aforementioned outcomes indicated that T4 alleviated TBI-induced brain histopathology, including BBB impairment and cerebral edema.

### Behavioral outcomes

**Fig. 6:**
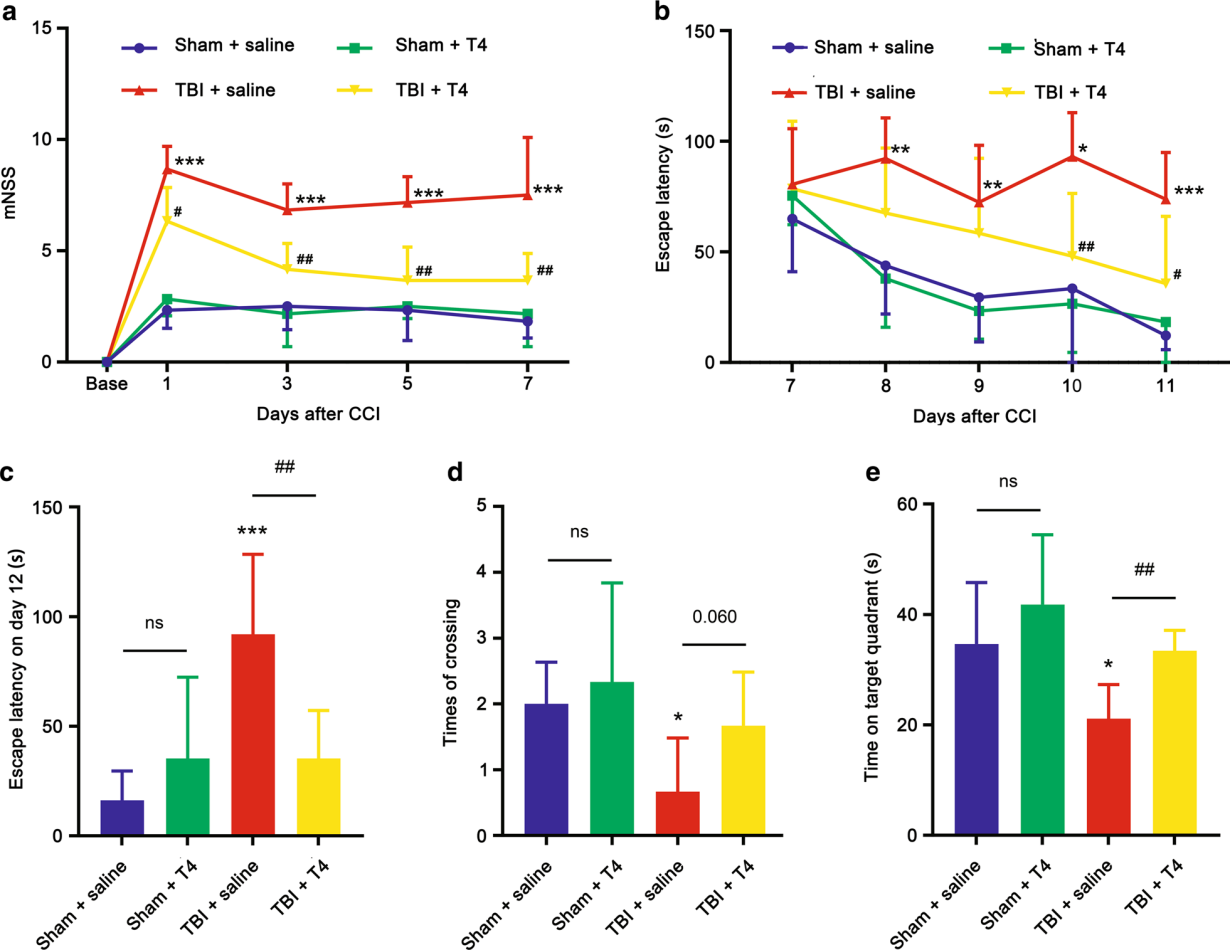
Behavioral outcomes. **a** mNSS score on days 1, 3, 5, and 7 post-TBI (n = 6 per group). **b** Escape latency in MWM test 7 to 11 days post-TBI (n = 6 per group). On test day (day 12), **c** escape latency, **d** number of platform crossings, and **e** time spent in platform quadrant were detected (n = 6 per group; *p < 0.05, **p < 0.01, and ***p < 0.001 vs. sham + saline; ^#^*p* < 0.05 and ^##^*p* < 0.01 vs. TBI + saline; ns: not significant). Error bars represent SD

To examine the impacts of T4 on improving behavioral performance and neurological function of rats post-CCI, mNSS and MWM tests were conducted (Fig. 1). As shown in Fig. 6a, on days 1, 3, 5, and 7 post-CCI, mNSS of the TBI + saline group was significantly higher than the sham + saline groups (*p* < 0.0001 in all groups). On days 1 and 3, mNSS of the TBI + T4 group was also significantly higher than the sham + saline group (*p* = 0.0002 on day 1 and *p* = 0.026 on day 3). However, on day 5, the difference between the two groups was not significant (*p* = 0.14). Additionally, significant differences were observed between the TBI + saline and TBI + T4 groups on all four days (*p* = 0.011 on day 1, *p* < 0.01 on days 3, 5, and 7).

The MWM test was performed at the beginning of day 7 (Fig. 6b). On day 7, differeces of escape latency among the four groups were not significant. After day 8, escape latency of the TBI + T4 group dropped gradually, and the TBI + saline group maintained a level higher than the sham + saline group (*p* = 0.0020 on day 8, *p* = 0.0090 on day 9, *p* = 0.0120 on day 10, and *p* < 0.0001 on day 11). After day 10, a significant difference between the TBI + saline and TBI + T4 groups was detected (*p* = 0.0098 on day 10 and *p* = 0.030 on day 11). On test day (day 12), escape latency of the TBI + saline group significantly increased compared to the sham + saline group (92.00 ± 36.39 vs. 16.18 ± 13.46, *p* = 0.0007). After treatment with T4, escape latency dropped dramatically (35.30 ± 21.85, *p* = 0.0084).

On day 12 post-CCI, the number of times rats crossed the location where the platform was previously placed was tested. As shown in Fig. 6d, rats in the TBI + saline group crossed the location less frequently than the sham + saline group (0.67 ± 0.82 vs. 2.00 ± 0.63 *p* = 0.010), and the TBI + T4 group crossed more frequently than the TBI + saline group (1.667 ± 0.82 *p* = 0.060). Time spent in the platform quadrant on test day was also measured (Fig. 6e), and the TBI + saline group spent significantly less time in the quadrant than the sham + saline group (21.15 ± 6.16 vs. 34.65 ± 11.15, *p* = 0.027) and TBI + T4 group (33.42 ± 3.706, *p* = 0.0019). Furthermore, difference in escape latency between the TBI + saline and sham + saline groups on test day was also significant (*p* = 0.0007). The effects of T4 on behavioral outcome improvements were evident.

### Proteomic analysis after T4 treatment

**Fig. 7:**
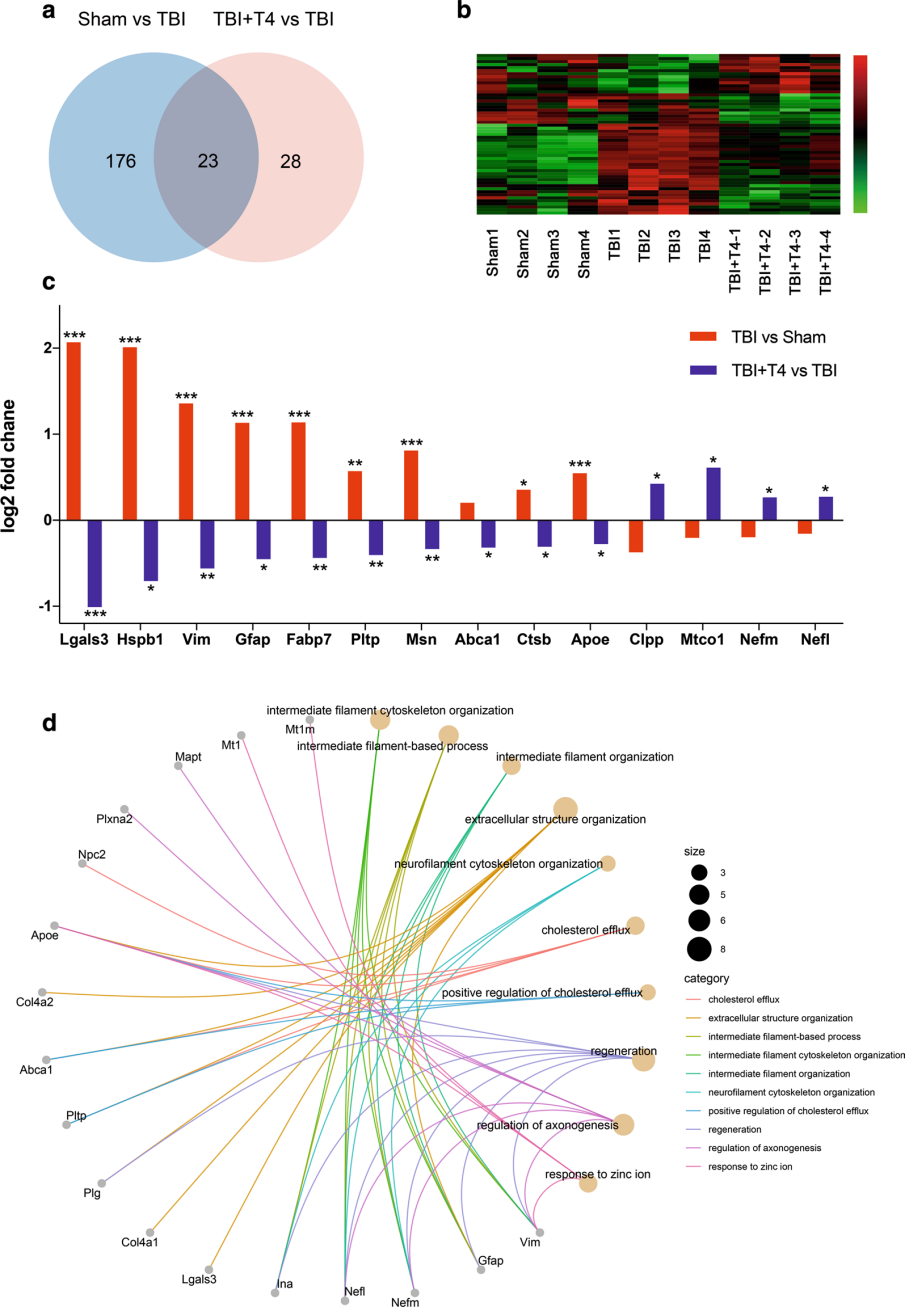
Proteomic analysis and GO enrichment after TBI and T4 treatment. **a** Venn diagram, TBI vs. sham and TBI + T4 vs. TBI show overlap in differentially expressed proteins. **b** Heatmap shows 51 proteins significantly changed after T4 treatment. The number in the color scale indicates z-score.** c** Examples of proteins significantly changed after T4 treatment.** d** Top 10 GO terms and proteins associated with these terms. Size of the circles represents number of proteins associated with the terms. (n = 4 per group; *p < 0.05, **p < 0.01, and ***p < 0.001)

We also conducted iTRAQ analysis of cortices after CCI and T4 treatment. Through whole proteomic profiling, a series of proteins and pathways potentially involved in T4 effects were identified. A total of 199 proteins were differentially expressed comparing sham + saline and TBI + saline groups and 51 differentially expressed proteins were identified comparing TBI + saline and TBI + T4 groups. Among these differential proteins, 23 proteins were altered by TBI and reversed by T4 treatment (Additional file 1: Table S1; Fig. 7a, b). Some proteins such as Lgals3, Hspb1, Vim, Gfap, Fabp7, Pltp, Msn, Ctsb, and Apoe were upregulated after TBI and reversed by T4 treatment, and the expression trend of proteins like Abca1, Clpp, Mtco1, Nefm and Nefl were significantly changed by T4 treatment (Fig. 7c). Most reversed proteins were upregulated after TBI and downregulated by T4 (Fig. 7c). The 51 proteins altered by T4 treatment were enriched for GO terms. Top 10 GO terms and genes associated with these terms were shown in Fig. 7d. Differential proteins were mostly associated with intermediate filament organization, cholesterol transportation and axonal regeneration.

## Discussion

Quantitative proteomics has been widely applied to the detection of differential proteins and pathways in many disease models, including TBI. In this study, an 8-plex iTRAQ analysis enabled the investigation of large-scale protein alterations. In total, 3937 proteins were identified, and 146 proteins were found to be differentially expressed. Some of these proteins have been discovered to be related to TBI. cAMP signaling pathway-related proteins, guanine nucleotide-binding protein G (Gnal) and phosphodiesterase 10A (Pde10a), were upregulated significantly in this study. KEGG also showed that the cAMP signaling pathway was significant. This was in agreement with a previous study by Song et al. [45]. This study further confirmd the relationship between cAMP-signaling pathway and TBI.

Recently, many studies have proven that Hsp70 increased rapidly in traumatically injured brains of mammals, including humans, and has protective effects on brain rehabilitation [54, 55]. This phenomenon was also reflected in the proteomic outcomes; although, no significant increase was detected in PRM. In addition, Hspb1 was upregulated in both iTRAQ and PRM, which has been associated with multiple sclerosis in the spinal cord [56]. Many proteins in the heat shock protein family have been found to be protective in neurodegenerative diseases and brain trauma [57, 58]; thus, further research is required to explore the effects of other proteins in this family on post-traumatic protection. Inconsistency between PRM and iTRAQ also exists. For example, Hsp70 upregulated eight folds in iTRAQ but did not significantly change in PRM. In proteomic analysis, fake positive is possible, because qualitative analysis of proteins is scored based on the matching degree and the number of peptides, the result is always a possibility. Also, Hsp70 has homologous proteins, which could affect the precision of identification. Thus, a verification like PRM becomes necessary. Upregulation of S100 calcium-binding protein A9 (S100A9) has been proposed to be related to acute neuroinflammation [54], which was consistent with the proteomic outcomes in this study. In addition, a recent study demonstrated that genetic deletion of S100A9 had a neuroprotective effect, which indicates that S100A9 may be a potential treatment target for brain trauma [59]. Also, Hpx protected BBB integrity in cerebral ischemia [60]. Gc is a binding protein and transports vitamin D, and vitamin D levels are significantly related to the severity of head injury. Vitamin D replacement after trauma may be beneficial [61]. Penk is a protein found in the endogenous opioid system and may be useful for predicting clinical outcomes of severe TBI, and it has been used to relieve craniofacial pain in rat models of TBI [62, 63].

The relationship between cocaine addiction and TBI was reflected by the KEGG results. Some previous research has reached similar conclusions. Individuals with cocaine addiction were found to have a higher proportion of TBI history than healthy individuals [64]. TBI has been proposed to augment the rewarding effects of some psychostimulants and has been linked with increased vulnerability to substance abuse [65, 66]. Drug addiction has been associated with dopamine, a neurotransmitter that controls motivation and reward [67]. This KEGG analysis also revealed that some upregulated proteins participated in dopaminergic synapse activity, and some proteins in this pathway (i.e., Camk2g, Ppp1r1b, Th, and Ddc) were also involved in cocaine or amphetamine addiction pathways. These results confirmed previous research findings that brain trauma may enhance drug addiction via dopamine synapse pathways. Interestingly, adrenergic signaling in cardiomyocytes and cardiac muscle contraction were related to TBI. Previous studies have illustrated that there were interactions between the heart and brain after TBI and that TBI may be a risk factor for myocardial dysfunction [68, 69].

Thyroid hormones have been proven essential for neuronal protection, recovery, and regeneration after brain trauma and are considered a potential therapeutic method [49, 51, 70]. Ttr, a major carrier of thyroid hormones that can cross the BBB and preferentially bind T4 [71], was shown to be upregulated post-TBI by iTRAQ analysis and verified by PRM. Recently, using unbiased single-cell sequencing, Arneson et al. demonstrated that upregulation of Ttr in different cell types in hippocampus after TBI and the use of T4 to modulate Ttr may mitigate behavioral outcomes and genetic problems caused by TBI in mice. The research concluded that upregulation of Ttr after TBI was due to a lack of thyroid hormones in the cerebrum and that T4 may reverse the deficiency. T4 treatment may reverse expression patterns of many T4 transporter genes, among which Ttr showed the most significant fold changes [72]. Our study confirmed that using T4 can ameliorate brain edema, improve integrity of the BBB, and improve cognitive outcome in TBI-affected rats. Some of the effects originated from the physiological functions of thyroxine itself, while some may be achieved by regulating Ttr. Another study also confirmed that Ttr increased as the severity of TBI strengthened; thus, Ttr may be used as a criterion to assess the severity of TBI [43]. The important role of Ttr in TBI has been further confirmed by quantitative proteomics in this study.

Proteomic profile between TBI and TBI + T4 revealed pathways responsible for T4 treatment. A total of 51 proteins differentially expressed after T4 treatment and 23 of the proteins reversed the alteration after TBI. Among the 23 proteins, many of them have been studied and shown to be associated with neuroinflammation, brain edema and BBB integrity. For example, Galectin-3 (Lgals3) is important in brain inflammatory response in neurodegenerative diseases [73, 74] and inhibiting galectin-3 has been proved to ameliorate brain edema in subarachnoid hemorrhage [75]. Hspb1 (Hsp27) responds to stress and has some neuroprotective effects. Activation of Hsp27 could ameliorate intracerebral hemorrhage-induced secondary brain injury and attenuate blood–brain barrier disruption [76, 77]. Vimentin and GFAP are intermediate filaments involved in neural plasticity and regeneration and play important roles in responses to stress such as injury, ischemia and neurodegeneration [78]. GFAP has also been confirmed to be a sensitive biomarker for TBI diagnosis and prediction, which was consistent to our proteomic outcome [79]. Fatty acid-binding protein-7 (Fabp7) in astrocytes was associated with glial differentiation, proliferation and neurogenesis, and has been confirmed to protect BBB integrity after TBI [80, 81]. Phospholipid transfer protein (PLTP) and moesin have been confirmed to be protective in BBB integrity by involving cerebrovascular remodeling and maintaining the function of transporters at BBB respectively [82, 83]. ATP-binding cassette transporter member A1 (ABCA1) and apolipoprotein E (ApoE) are major cholesterol transporters that play essential roles in cholesterol homeostasis in brain. ABCA1/ApoE were reported in many research about their effects in neural restoration after stroke [84]. Some of these upregulations after TBI could have protective effects and some were just reactive. With T4 treatment, possibly because the BBB integrity, vascular pathology and brain edema were alleviated, these upregulations were reversed. Some proteins downregulated after TBI but upregulated significantly with T4 treatment, although the downregulation after TBI was not significant in most proteins. Among these proteins, Nefm and Nefl were enriched in GO terms such as regeneration and axonogenesis, which probably indicated that T4 treatment contributes to neural regeneration. Cytochrome *c* oxidase (Mtco1) is an enzyme in the mitochondrial electron transport chain for oxidative phosphorylation [85], upregulation with T4 treatment suggested mitochondrial function was restored. Combining with GO enrichment, T4 treatment could ameliorate brain inflammation, edema and BBB damage and involves in neural regeneration, cholesterol transporting, intermediate filament organization and mitochondrial function pathways.

Based on iTRAQ proteomic profiling, a data set to summarize protein alterations after TBI was generated. The PRM method validated some proteins, such as Ttr, as reliable biomarkers or treatment targets for TBI. Proteomic analysis also revealed pathways and differential proteins involved in T4 treatment after TBI. iTRAQ proteomic technologies can effectively facilitate the exploration of important differential proteins and help detect disease mechanisms.

## Supplementary Information

**Additional file 1: Table S1.** Identified proteins and differentially expressed proteins in iTRAQ.

**Additional file 2: Table S2.** Quantitative data of peptides and proteins in PRM.

**Additional file 3: Figure S1.** Chromatograms of fragment ions and peak area of peptide FTEGVY corresponding to Ttr.

**Additional file 4: Figure S2.** Chromatograms of fragment ions and peak area of peptide GSPAVDVAVK corresponding to Ttr.

## Data Availability

All of the datasets in this study can be obtained by reasonable request to the corresponding authors.

## Abbreviations

iTRAQ: Isobaric tags for relative and absolute quantitation
LC–MS/MS: Liquid chromatography–tandem mass spectrometry
mNSS: Modified neurological severity scores
MWM: Morris water maze
PRM: Parallel reaction monitoring
RPLC: Reversed-phase liquid chromatography
T4: Thyroxine
TBI: Traumatic brain injury
Ttr: Transthyretin
