# Supplementary figures and images for: iTRAQ-based proteomic profiling reveals protein alterations after traumatic brain injury and supports thyroxine as a potential treatment
Source: Mol Brain. 2021 Jan 27;14:25. doi: 10.1186/s13041-021-00739-0 (PMC7839205; doi:10.1186/s13041-021-00739-0)

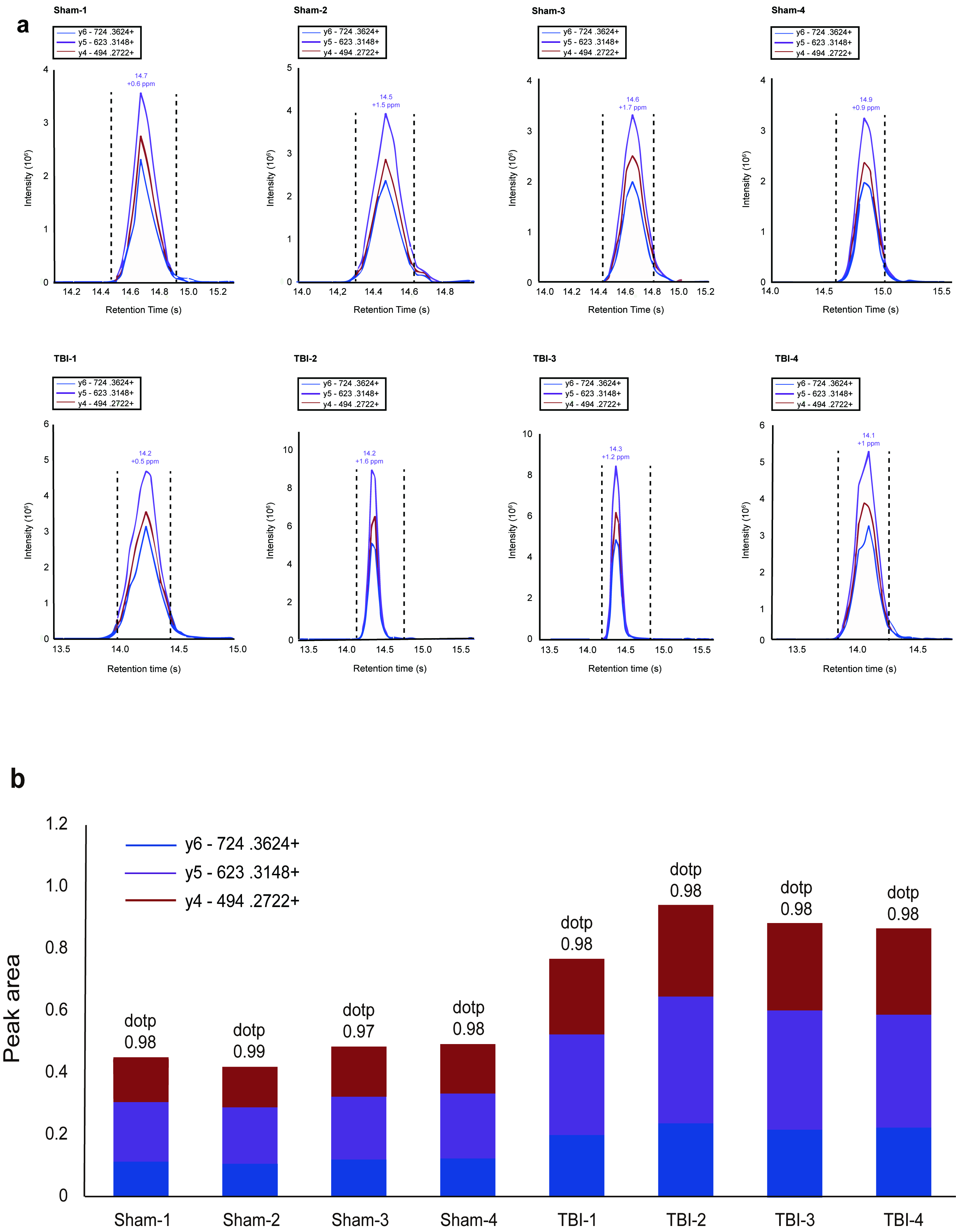

Supplement: Supplementary file 3 — Additional file 3: Figure S1. Chromatograms of fragment ions and peak area of peptide FTEGVY corresponding to Ttr. [file 13041_2021_739_MOESM3_ESM.tif]

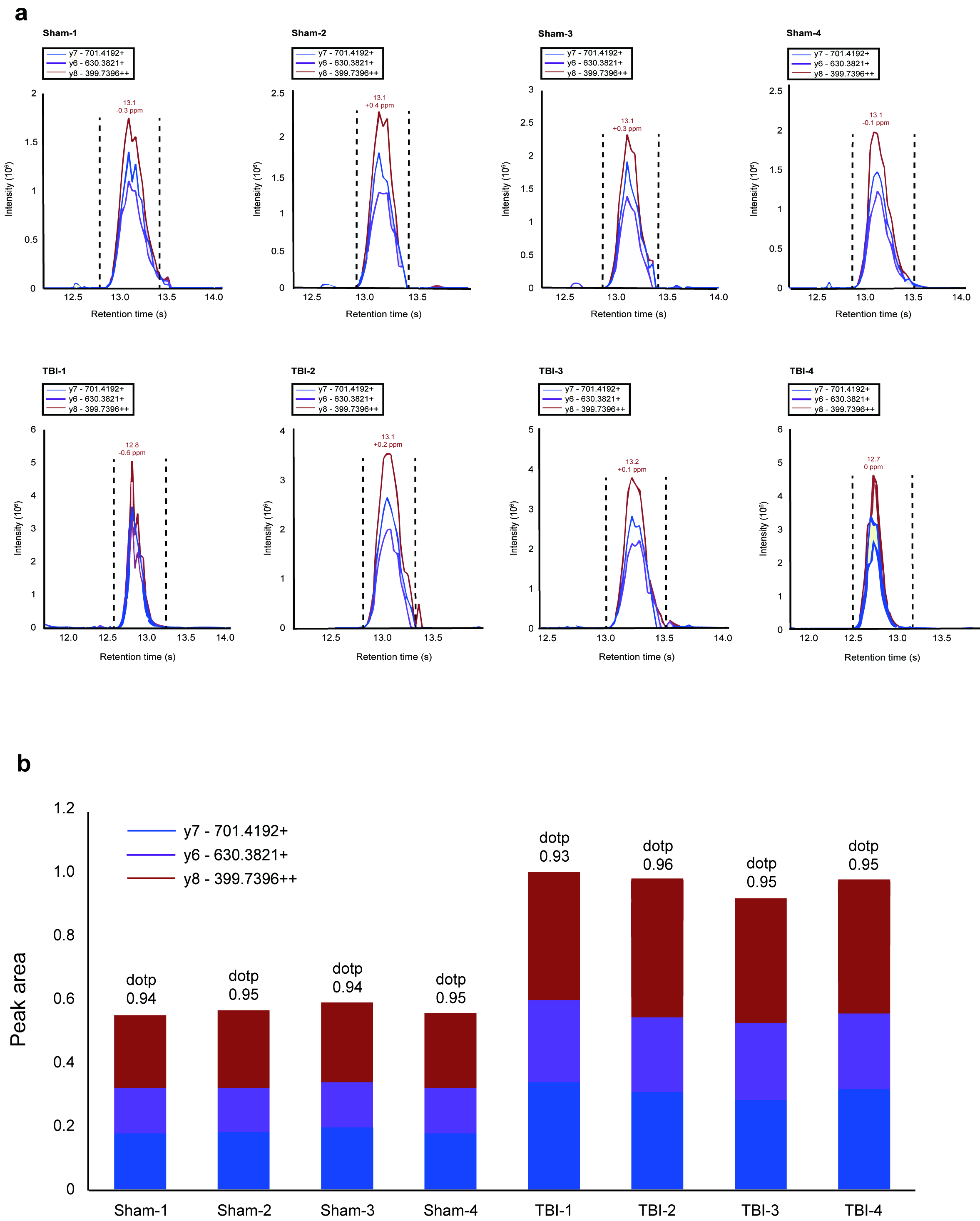

Supplement: Supplementary file 4 — Additional file 4: Figure S2. Chromatograms of fragment ions and peak area of peptide GSPAVDVAVK corresponding to Ttr. [file 13041_2021_739_MOESM4_ESM.tif]
